# Frequency of nut consumption and mortality risk in the PREDIMED nutrition intervention trial

**DOI:** 10.1186/1741-7015-11-164

**Published:** 2013-07-16

**Authors:** Marta Guasch-Ferré, Mònica Bulló, Miguel Ángel Martínez-González, Emilio Ros, Dolores Corella, Ramon Estruch, Montserrat Fitó, Fernando Arós, Julia Wärnberg, Miquel Fiol, José Lapetra, Ernest Vinyoles, Rosa Maria Lamuela-Raventós, Lluís Serra-Majem, Xavier Pintó, Valentina Ruiz-Gutiérrez, Josep Basora, Jordi Salas-Salvadó

**Affiliations:** 1 Human Nutrition Unit, Hospital Universitari de Sant Joan de Reus, Faculty of Medicine and Health Sciences, IISPV (Institut d’Investigació Sanitària Pere Virgili), Universitat Rovira i Virgili, Reus, Spain; 2 CIBERobn (Centro de Investigación Biomédica en Red Fisiopatología de la Obesidad y Nutrición), Institute of Health Carlos III, Madrid, Spain; 3 Department of Preventive Medicine and Public Health, University of Navarra, Pamplona, Spain; 4 Lipid Clinic, Department of Endocrinology and Nutrition, Institut d’Investigacions Biomediques August Pi Sunyer (IDIBAPS), Hospital Clinic, University of Barcelona, Barcelona, Spain; 5 Department of Preventive Medicine, University of Valencia, València, Spain; 6 Department of Internal Medicine, Institut d’Investigacions Biomèdiques August Pi Sunyer (IDIBAPS), Hospital Clínic, University of Barcelona, Barcelona, Spain; 7 Cardiovascular Risk and Nutrition Research Group, Institut Hospital del Mar d’Investigacions Mèdiques (IMIM), Barcelona Biomedical Research Park, Barcelona, Spain; 8 Department of Cardiology, University Hospital Txagorritxu, Vitoria, Spain; 9 Department of Preventive Medicine, University of Malaga, Malaga, Spain; 10 Institute of Health Sciences, University of Balearic Islands and Hospital Son Espases, Palma de Mallorca, Spain; 11 Department of Family Medicine, Primary Care Division of Sevilla, San Pablo Health Center, Sevilla, Spain; 12 Primary Health Care Division and Research, Instituto de Investigaciones Biomédicas August Pi i Sunyer-Jordi Gol, Barcelona, Spain; 13 INSA, University of Barcelona, Barcelona, Spain; 14 Department of Clinical Sciences, University of Las Palmas de Gran Canaria, Las Palmas, Spain; 15 Lipids and Vascular Risk Unit, Internal Medicine, Hospital Universitario de Bellvitge, Hospitalet de Llobregat, Barcelona, Spain; 16 Instituto de la Grasa, Consejo Superior de Investigaciones Científicas, Sevilla, Spain

**Keywords:** Cancer, Cardiovascular, Mortality, Nuts, PREDIMED study

## Abstract

**Background:**

Prospective studies in non-Mediterranean populations have consistently related increasing nut consumption to lower coronary heart disease mortality. A small protective effect on all-cause and cancer mortality has also been suggested. To examine the association between frequency of nut consumption and mortality in individuals at high cardiovascular risk from Spain, a Mediterranean country with a relatively high average nut intake per person.

**Methods:**

We evaluated 7,216 men and women aged 55 to 80 years randomized to 1 of 3 interventions (Mediterranean diets supplemented with nuts or olive oil and control diet) in the PREDIMED (‘PREvención con DIeta MEDiterránea’) study. Nut consumption was assessed at baseline and mortality was ascertained by medical records and linkage to the National Death Index. Multivariable-adjusted Cox regression and multivariable analyses with generalized estimating equation models were used to assess the association between yearly repeated measurements of nut consumption and mortality.

**Results:**

During a median follow-up of 4.8 years, 323 total deaths, 81 cardiovascular deaths and 130 cancer deaths occurred. Nut consumption was associated with a significantly reduced risk of all-cause mortality (*P* for trend <0.05, all). Compared to non-consumers, subjects consuming nuts >3 servings/week (32% of the cohort) had a 39% lower mortality risk (hazard ratio (HR) 0.61; 95% CI 0.45 to 0.83). A similar protective effect against cardiovascular and cancer mortality was observed. Participants allocated to the Mediterranean diet with nuts group who consumed nuts >3 servings/week at baseline had the lowest total mortality risk (HR 0.37; 95% CI 0.22 to 0.66).

**Conclusions:**

Increased frequency of nut consumption was associated with a significantly reduced risk of mortality in a Mediterranean population at high cardiovascular risk.

Please see related commentary: http://www.biomedcentral.com/1741-7015/11/165.

**Trial registration:**

Clinicaltrials.gov. International Standard Randomized Controlled Trial Number (ISRCTN): 35739639. Registration date: 5 October 2005.

## Background

Nuts are an important component of the so-called Mediterranean diet (MedDiet) and a good source of unsaturated fatty acids, fiber, minerals (potassium, calcium and magnesium), vitamins (folate and tocopherols) and other bioactive compounds, such as phytosterols and polyphenols [1]–[4].

There is consistent evidence to suggest that the consumption of nuts has a beneficial effect on cardiovascular health, and this effect is attributable to their unique nutritional composition [5]. A pooled analysis of four large-scale observational studies showed that subjects in the highest nut consumption categories had an approximately 35% reduced risk of incident coronary heart disease (CHD) [6]. The frequency of nut consumption was also related to lower rates of sudden cardiac death in a large cohort of men [7]. Furthermore, epidemiologic studies and clinical trials have shown that frequent nut consumption is associated with a reduced load of cardiovascular disease risk factors, such as dyslipidemia, type 2 diabetes, and metabolic syndrome [4,6,8,9]. In addition, reports from the Iowa Women’s Health study [10], a large Dutch cohort [11], and the US Nurses’ Health Study [12], which assessed populations with relatively low overall nut intake, suggested that frequent nut consumption related inversely to total mortality, albeit the protective effect was weak, with adjusted risk reductions ranging from 5% to 15% [10]–[12]. If an inverse association between nut consumption and all-cause mortality exists, the beneficial effect might be more robust in Mediterranean regions, where nut consumption per person is relatively high compared to other countries [13].

The main aim of the PREDIMED study was to test the efficacy of two Mediterranean diets (one supplemented with extra-virgin olive oil and another with nuts), as compared to a control diet (advice on a low-fat diet), on primary cardiovascular prevention. In contrast, in this current manuscript our aims were only to assess the association between baseline consumption of nuts (that is, the consumption of nuts previous to starting the intervention) and total mortality (instead of cardiovascular events). We have additionally included the repeated measurements for the consumption of nuts during follow-up as another exposure, regardless of the allocated arm of the trial; this is in contrast with the original PREDIMED study, which used an intention-to-treat analysis.

We hypothesized that level of nut consumption would be strongly associated with mortality in the cohort of the PREDIMED (‘PREvención con DIeta MEDiterránea’) study, including older men and women at high cardiovascular risk [14]. To this end, in this cohort we longitudinally examined the association between the frequency of nut consumption at baseline and the risk of mortality at the end of follow-up.

## Methods

### Study population

The present study was conducted within the framework of the PREDIMED trial, the design of which has been described in detail elsewhere [14]. Briefly, the PREDIMED study is a large, multicenter, parallel-group, randomized and controlled clinical trial for the primary prevention of cardiovascular disease (CVD) (http://www.predimed.es and http://www.predimed.org). The main results of the trial on the primary endpoint have been recently published [15]. We assigned 7,447 older participants (men aged 55 to 80 years and women 60 to 80 years) to 1 of 3 interventions: a MedDiet enriched with extra-virgin olive oil (EVOO), a MedDiet supplemented with mixed nuts, or advice on a low-fat diet (control diet). Participants had no CVD at enrollment but they were at high cardiovascular risk because of the presence of type 2 diabetes or at least three of the following risk factors: current smoking, hypertension, hypercholesterolemia, low high-density lipoprotein (HDL)-cholesterol, overweight or obesity, and family history of premature CVD. Exclusion criteria were the presence of severe medical condition that may impair the ability of the person to participate in a nutrition intervention study (for example, digestive disease with fat intolerance, advanced malignancy, or major neurological, psychiatric or endocrine disease), immunodeficiency or HIV positive status, alcohol or drug abuse, body mass index (BMI) ≥40 kg/m^2^, and allergy or intolerance to olive oil or nuts [16].

The primary endpoint of the main trial is a combination of several cardiovascular events (myocardial infarction, stroke or cardiovascular death). The present study was conducted as an observational cohort using baseline consumption of nuts as the exposure. The outcomes were: (1) total mortality, (2) only cardiovascular mortality, and (3) only cancer mortality. All participants provided written informed consent according to a protocol approved by the institutional review boards of the recruiting centers (Comité de Ética e Investigación Clínica (CEIC) Hospital Universitari Sant Joan de Reus, CEIC Universidad de Navarra, CEIC Hospital Clínic de Barcelona, Comité de Ética Universidad de Valencia, CEIC-Parc de Salut Mar, CEIC Hospital Universitario Araba, CEIS del distrito Sanitario Atención Primária Sevilla, IDIAP Jordi Gol, CEIC Complejo Hospitalario Materno-Insular, CEIC Facultad Medicina Universidad de Málaga, CEIC Illes Balears, and CEIC Hospital Universitari Bellvitge).

### Dietary assessment

At baseline trained dietitians completed a 137-item semiquantitative food frequency questionnaire in a face-to-face interview with the participant; this questionnaire has been validated before in an older population at high cardiovascular risk from Spain [17]. Energy and nutrient intake were estimated using Spanish food composition tables [18,19]. Information on self-reported nut intake was derived from the food frequency questionnaire. The questionnaire includes one item regarding the consumption of almonds, peanuts, hazelnuts, pistachios and pine nuts (macadamias, cashews and Brazil nuts are rarely consumed in Spain), and another question specifically inquired about the consumption of walnuts. The dietitians asked the participants if they consumed this food item never, between 1 to 3 times per month, times per week (1, 2 to 4, 5 to 6; three options) or times a day (1, 2 to 3, 4 to 6, >6; four options). For the purpose of the present study, 28 g of nuts was considered to be one serving. Peanuts, almonds, hazelnuts, walnuts, pine nuts, pistachios, Brazil nuts, macadamia and cashews were all considered nuts. In addition, dietitians administered a validated 14-item MedDiet screener designed to assess the degree of adherence to the traditional MedDiet [20]. We used the score of this brief screener to control for the overall dietary pattern, because a higher adherence to the MedDiet among frequent consumers of nuts could introduce confounding. For this purpose, the question about nut consumption was omitted from the brief screener; therefore, a 13-point score was used as a covariate (minimum 0, maximum 13).

### Ascertainment of mortality

Information on mortality was updated once a year by the End-point Adjudication Committee, whose members were blinded to treatment allocation. Different sources of information were used: (1) yearly questionnaires and examinations to all participants, (2) family physicians, (3) yearly review of medical records, and (4) linkage to the National Death Index. Medical records of deceased participants were requested, and the End-point Adjudication Committee adjudicated the cause of the death.

### Assessment of other covariates

At baseline, questionnaires about lifestyle variables, educational achievement, history of illnesses, and medication use were administered. Physical activity was assessed using the validated Spanish version of the Minnesota Leisure-Time Physical Activity questionnaire [21]. Participants were considered to be diabetic, hypercholesterolemic or hypertensive if they had previously been diagnosed as such, and/or they were being treated with antidiabetic, cholesterol-lowering, or antihypertensive agents, respectively. Trained personnel took the anthropometric and blood pressure measurements. Weight and height were measured with light clothing and no shoes with calibrated scales and a wall-mounted stadiometer, respectively; waist circumference was measured midway between the lowest rib and the iliac crest using an anthropometric tape; blood pressure was measured using a validated oscillometer (Omron HEM705CP; Hoofddorp, The Netherlands) in triplicate with a 5-minute interval between each measurement, and the mean of these values was recorded.

### Statistical analyses

Follow-up time was calculated as the difference between the date of either death or end of follow-up (the date of the last visit or the last recorded clinical event of participants still alive) and the date of recruitment. Extremes of total energy intake (>4,000 or <800 kcal per day in men and >3,500 or <500 kcal per day in women) were excluded from the analysis [22]. Three categories of frequency of nut consumption were considered (never or almost never, 1 to 3 servings per week and >3 servings per week). We used analysis of variance (ANOVA) or the Pearson χ^2^ tests to compare the quantitative or categorical baseline characteristics of the study participants, respectively, across servings of nut consumption. Results were expressed as means ± SD or percentages. Because no interaction was observed between sex and the main outcome, analyses were conducted for men and women together.

To assess the risk of total mortality by frequency of nut consumption, multivariate relative risks were computed using Cox proportional hazard models, and potential confounders were controlled for. All analyses were stratified by the recruitment center. Results are expressed as hazard ratios (HRs) with 95% confidence intervals (CIs). Given the different nutritional composition of walnuts and other nuts [1], we performed separate analyses for the frequency of total nut consumption, walnut consumption, and consumption of nuts excluding walnuts. After the unadjusted model, another model was adjusted for age (continuous), sex and intervention group. Then, a second model, was additionally adjusted for BMI (continuous), current smoking status (never, former, or current smoker), educational level (illiterate/primary education, secondary education, academic/graduate), physical activity (MET-min/day), total energy intake (kcal/day), history of diabetes (yes/no), history of hypercholesterolemia (yes/no), use of oral antidiabetic medication (yes/no), antihypertensive drugs (yes/no), and statins (yes/no). Finally, a third, fully-adjusted model, was additionally adjusted for alcohol intake (continuous, adding a quadratic term), quintiles of consumption of dietary food groups (vegetables, fruits, red meat, eggs, and fish), and adherence to the MedDiet (13-point score). The same models were used to assess the risk of cardiovascular mortality or cancer mortality, also using Cox proportional hazard models. Linear trend tests were assessed assigning the median value to each category of nut consumption and using it as a continuous variable in the various models. We evaluated the interaction between baseline nut consumption (three categories, two dummy variables) and the intervention group (three groups, two dummy variables) by introducing an interaction term with four degrees of freedom in the model. We used Cox regression models to assess the risk of total mortality, cardiovascular mortality and cancer mortality according to the joint categories of total nut consumption and intervention group. Linear trends were also tested. We had yearly updated information on nut consumption, so to take advantage of this updated information we repeated the analysis using generalized estimating equations to assess the association between repeated measurements of nut consumption and mortality. For each 1-year period we used as exposure the average nut consumption of all repeated measurements from baseline to the beginning of that yearly period.

The level of significance for all statistical tests was *P* <0.05 for bilateral contrast. Analyses were performed using SPSS statistical software, version 19 (SPSS Inc, Chicago, IL, USA) and STATA software, version 12.0 (Stata Corp., College Station, TX, USA).

## Results

After those subjects with extremes of total energy intake (n = 153) and those with incomplete dietary data (lack of food frequency questionnaire) at baseline (n = 78) had been excluded, 7,216 individuals were available for the present analysis. The mean age of the participants was 67 years and there were a total of 3,071 men and 4,145 women. Table 1 shows the baseline characteristics of study participants by frequency of total nut consumption. Subjects who ate nuts more frequently had lower BMI and waist circumference, were less likely to smoke, and were more physically active compared to those who rarely or never consumed nuts. In the upper category of nut consumption there were fewer individuals with type 2 diabetes mellitus or who used antidiabetic and antihypertensive medication. In addition, frequent nut consumption was associated with a higher intake of energy, vegetables, fruit, and fish.

**Table 1:** Baseline characteristics of study participants by frequency of nut consumption

**Variable**	**Baseline frequency of nut consumption**			
	**Never (n = 2,118)**	**1 to 3 servings/week (n = 2,803)**	**>3 servings/week (n = 2,295)**	***P *** **value**
Age, years	67 ± 6	66 ± 6	67 ± 6	<0.001
Men, % (n)	36 (773)	43 (1,219)	47 (1,079)	<0.001
BMI, kg/m^2^	30.6 ± 4.0	29.9 ± 3.7	29.4 ± 3.7	<0.001
Weight, kg	77.2 ± 12.0	77.1 ± 12.1	75.8 ± 11.6	<0.001
Waist circumference, cm	101.5 ± 10.5	100.7 ± 10.4	99.2 ± 10.0	<0.001
Leisure-time energy expenditure in physical activity, MET-min/day	195 ± 220	231 ± 232	264 ± 257	<0.001
Smoking status, % (n)				<0.001
Never	64 (1,367)	61 (1,708)	59 (1,364)	
Current	14 (310)	14 (402)	12 (292)	
Former	21 (441)	24 (693)	28 (639)	
Educational level, % (n)				<0.001
Illiterate/primary education	82 (1,740)	76 (2,131)	75 (1,733)	
Secondary education	12 (266)	16 (451)	16 (379)	
Academic/graduate	5 (112)	8 (221)	8 (183)	
Diabetes, % (n)	53 (1,118)	47 (1,338)	46 (1,071)	<0.001
Hypertension, % (n)	83 (1,763)	83 (2,320)	82 (1,887)	0.670
Hypercholesterolemia, % (n)	70 (1,479)	73 (2,044)	73 (1,689)	0.012
Medication use, % (n)				
Oral antidiabetic drugs	36 (760)	31 (884)	29 (679)	<0.001
Antihypertensive drugs	75 (1,587)	72 (2,037)	71 (1,624)	0.008
Statins	48 (1,022)	48 (1,364)	47 (1,093)	0.761
Modified MedDiet score (13-point score)	8.1 ± 1.7	8.2 ± 1.8	8.6 ± 1.7	<0.001
Total energy intake, g/day	2,060 ± 529	2,222 ± 514	2,416 ± 537	<0.001
Nuts, g/day	0	4.9 ± 2.3	25.7 ± 14.4	<0.001
Alcohol, g/day	6.6 ± 13.4	8.4 ± 13.8	9.7 ± 14.7	<0.001
Vegetables, g/day	317 ± 144	329 ± 145	355 ± 149	<0.001
Fruit, g/day	344 ± 200	354 ± 195	407 ± 204	<0.001
Red meat (beef, pork, lamb), g/day	70.4 ± 44.3	79.1 ± 46.1	78.6 ± 46.2	<0.001
White meat (chicken, rabbit, turkey), g/day	44.6 ± 28.5	45.1 ± 27.1	44.5 ± 27.6	0.720
Eggs, g/day	19.4 ± 10.9	20.4 ± 11.5	20.0 ± 10.6	0.010
Fish, g/day	91.8 ± 47.8	99.9 ± 48.9	105.2 ± 53.7	<0.001
Dairy products, g/day	387 ± 224	374 ± 216	381 ± 222	0.095

Data are expressed as mean ± SD or percentage (n). *P* value for comparisons across servings of nut consumption (Pearson χ^2^ test for categorical variables or one-way analysis of variance for continuous variable) as appropriate.

BMI, body mass index.

Changes in total nut consumption were +15.95 ± 21.10 g/day (mean ± SD) in the MedDiet supplemented with nuts, -0.80 ± 16.31 g/day in the MedDiet supplemented with extra virgin olive oil and −3.12 ± 13.85 g/day in the control group.

During a median follow-up of 4.8 years, 323 total deaths, 81 cardiovascular deaths and 130 cancer deaths occurred. Table 2 shows HRs for total mortality by frequency of total nut consumption, walnut consumption, and consumption of other nuts. After adjustments for age, sex and intervention group (model 1), the subjects who ate nuts more frequently had a lower risk of total mortality in all the types of nuts analyzed (*P* for trend <0.001 for total nut and walnut consumption, and *P* = 0.010 for non-walnut nuts). In fully adjusted models, participants who consumed total nuts, walnuts, or non-walnut nuts >3 servings per week had significant reductions in total mortality risk of 39%, 45%, and 34%, respectively, compared to those who rarely or never consumed nuts. The relationship between nut consumption and total mortality was linear for all the models (*P* for trend <0.05), except for the crude model of nut consumption excluding walnuts.

**Table 2:** Hazard ratios of total mortality according to the frequency of nut consumption (including and not including walnuts)

**Total mortality**	**Never**	**1 to 3 servings/week**	**>3 servings/week**	***P *** **for trend**
Frequency of total nut consumption:	n = 2,118	n = 2,803	n = 2,295	
All causes of death, % (n)	5.6 (119)	4.2 (117)	3.8 (87)	
Person-years, n	8,724	12,168	10,185	
Crude model	1 (Reference)	0.68 (0.52 to 0.88)	0.60 (0.45 to 0.79)	0.005
Multivariable model 1	1 (Reference)	0.68 (0.52 to 0.89)	0.55 (0.41 to 0.73)	0.001
Multivariable model 2	1 (Reference)	0.69 (0.53 to 0.91)	0.59 (0.43 to 0.79)	0.005
Multivariable model 3	1 (Reference)	0.71 (0.54 to 0.93)	0.61 (0.45 to 0.83)	0.012
Frequency of walnut consumption:	n = 2,916	n = 2,547	n = 1,753	
All causes of death, % (n)	5.6 (164)	3.9 (100)	3.4 (59)	
Person-years, n	12,124	11,122	7,825	
Crude model	1 (Reference)	0.64 (0.50 to 0.83)	0.54 (0.40 to 0.73)	<0.001
Multivariable model 1	1 (Reference)	0.66 (0.51 to 0.85)	0.50 (0.37 to 0.68)	<0.001
Multivariable model 2	1 (Reference)	0.65 (0.50 to 0.84)	0.53 (0.39 to 0.73)	<0.001
Multivariable model 3	1 (Reference)	0.66 (0.51 to 0.86)	0.55 (0.40 to 0.76)	<0.001
Frequency of consumption of other nuts (excluding walnuts):	n = 3,308	n = 2,643	n = 1,265	
All causes of death, % (n)	5.0 (166)	4.1 (109)	3.8 (48)	
Person-years, n	13,936	11,573	5,566	
Crude model	1 (Reference)	0.77 (0.60 to 0.98)	0.71 (0.52 to 0.98)	0.068
Multivariable model 1	1 (Reference)	0.75 (0.59 to 0.96)	0.62 (0.44 to 0.86)	0.010
Multivariable model 2	1 (Reference)	0.78 (0.61 to 1.00)	0.64 (0.45 to 0.90)	0.021
Multivariable model 3	1 (Reference)	0.80 (0.62 to 1.03)	0.66 (0.46 to 0.93)	0.031

One serving of nuts equals 28 g. Cox regression models were used to assess the risk of all-cause mortality by frequency of nut consumption. Multivariable model 1 was adjusted for age in years, sex, and intervention group. Model 2 was additionally adjusted for body mass index (BMI) in kg/m^2^, smoking status (never, former, current smoker), educational level (illiterate/primary education, secondary education, academic/graduate), leisure time physical activity in MET-min/day, history of diabetes (yes/no), history of hypercholesterolemia (yes/no), use of oral antidiabetic medication (yes/no), use of antihypertensive medication (yes/no), use of statins (yes/no), and total energy intake (kcal/day). Model 3 was also adjusted for dietary variables in quintiles (vegetables, fruits, red meat, eggs, and fish), alcohol intake (continuous, adding a quadratic term) and Mediterranean diet adherence (13-point score). All models were stratified by recruitment centre. Extremes of total energy intake were excluded.

Those participants who ate total nuts, walnuts or other nuts (excluding walnuts) >3 servings per week had also lower risk of cardiovascular mortality than those in the reference category (Table 3). The HR of cardiovascular mortality in the fully-adjusted model of total nut consumption was 0.45 (95% CI 0.25 to 0.81). Table 4 shows the HRs for cancer death by frequency of total nut consumption, walnut consumption and consumption of other nuts. Subjects in the upper category of total nut consumption had a significant 40% (95% CI −37% to −98%) reduction in cancer death, although the *P* for trend was non significant.

**Table 3:** Hazard ratios of cardiovascular mortality according to the frequency of nut consumption (including and not including walnuts)

**Cardiovascular mortality**	**Never**	**1 to 3 servings/week**	**>3 servings/week**	***P *** **for trend**
Frequency of total nut consumption:	n = 2,118	n = 2,803	n = 2,295	
Cardiovascular death, % (n)	1.7 (36)	0.8 (23)	1.0 (22)	
Person-years, n	8,724	12,168	10,185	
Crude model	1 (Reference)	0.44 (0.26 to 0.74)	0.50 (0.29 to 0.85)	0.101
Multivariable model 1	1 (Reference)	0.44 (0.26 to 0.76)	0.47 (0.27 to 0.82)	0.075
Multivariable model 2	1 (Reference)	0.41 (0.24 to 0.71)	0.41 (0.23 to 0.73)	0.042
Multivariable model 3	1 (Reference)	0.42 (0.24 to 0.74)	0.45 (0.25 to 0.81)	0.091
Frequency of walnut consumption:	n = 2,916	n = 2,547	n = 1,753	
Cardiovascular death, % (n)	1.6 (46)	0.7 (18)	1.0 (17)	
Person-years, n	12,124	11,122	7,825	
Crude model	1 (Reference)	0.41 (0.24 to 0.71)	0.55 (0.31 to 0.96)	0.037
Multivariable model 1	1 (Reference)	0.42 (0.24 to 0.74)	0.54 (0.30 to 0.95)	0.040
Multivariable model 2	1 (Reference)	0.39 (0.22 to 0.69)	0.49 (0.27 to 0.88)	0.022
Multivariable model 3	1 (Reference)	0.41 (0.23 to 0.73)	0.53 (0.29 to 0.98)	0.047
Frequency of consumption of other nuts (excluding walnuts):	n = 3,308	n = 2,643	n = 1,265	
Cardiovascular death, % (n)	1.3 (43)	1.1 (28)	0.8 (10)	
Person-years, n	13,936	11,573	5,566	
Crude model	1 (Reference)	0.76 (0.47 to 1.22)	0.57 (0.28 to 1.13)	0.129
Multivariable model 1	1 (Reference)	0.73 (0.45 to 1.20)	0.48 (0.23 to 0.97)	0.056
Multivariable model 2	1 (Reference)	0.70 (0.43 to 1.15)	0.40 (0.19 to 0.83)	0.021
Multivariable model 3	1 (Reference)	0.74 (0.45 to 1.23)	0.42 (0.20 to 0.89)	0.031

One serving of nuts equals 28 g. Cox regression models were used to assess the risk of cardiovascular mortality by frequency of nut consumption. Multivariable model 1 was adjusted for age (years), sex, and intervention group. Model 2 was additionally adjusted for BMI (kg/m^2^), smoking status (never, former, current smoker), educational level (illiterate/primary education, secondary education, academic/graduate), leisure time physical activity (MET-min/day), history of diabetes (yes/no), history of hypercholesterolemia (yes/no), use of oral antidiabetic medication (yes/no), use of antihypertensive medication (yes/no), use of statins (yes/no), and total energy intake (kcal/day). Model 3 was also adjusted for dietary variables in quintiles (vegetables, fruits, red meat, eggs, and fish), alcohol intake (continuous, adding a quadratic term) and Mediterranean diet adherence (13-point score). All models were stratified by recruitment centre. Extremes of total energy intake were excluded.

**Table 4:** Hazard ratios of cancer mortality according to the frequency of nut consumption (including and not including walnuts)

**Cancer mortality**	**Never**	**1 to 3 servings/week**	**>3 servings/week**	***P *** **for trend**
Frequency of total nut consumption:	n = 2,118	n = 2,803	n = 2,295	
Cancer death, % (n)	2.1 (44)	1.9 (52)	1.5 (34)	
Person-years, n	8,724	12,168	10,185	
Crude model	1 (Reference)	0.82 (0.55 to 1.23)	0.64 (0.41 to 1.00)	0.070
Multivariable model 1	1 (Reference)	0.77 (0.51 to 1.16)	0.54 (0.34 to 0.86)	0.015
Multivariable model 2	1 (Reference)	0.79 (0.52 to 1.20)	0.60 (0.37 to 0.96)	0.052
Multivariable model 3	1 (Reference)	0.79 (0.52 to 1.20)	0.60 (0.37 to 0.98)	0.064
Frequency of walnut consumption:	n = 2,916	n = 2,547	n = 1,753	
Cancer death, % (n)	2.1 (62)	1.9 (48)	1.1 (20)	
Person-years, n	12,124	11,122	7,825	
Crude model	1 (Reference)	0.82 (0.56 to 1.20)	0.48 (0.29 to 0.80)	0.005
Multivariable model 1	1 (Reference)	0.76 (0.52 to 1.12)	0.41 (0.25 to 0.69)	0.001
Multivariable model 2	1 (Reference)	0.77 (0.52 to 1.14)	0.46 (0.27 to 0.77)	0.003
Multivariable model 3	1 (Reference)	0.76 (0.51 to 1.12)	0.46 (0.27 to 0.79)	0.005
Frequency of consumption of other nuts (excluding walnuts):	n = 3,308	n = 2,643	n = 1,265	
Cancer death, % (n)	2.0 (66)	1.6 (43)	1.7 (21)	
Person-years, n	13,936	11,573	5,566	
Crude model	1 (Reference)	0.77 (0.52 to 1.13)	0.79 (0.48 to 1.29)	0.439
Multivariable model 1	1 (Reference)	0.74 (0.50 to 1.10)	0.68 (0.41 to 1.14)	0.213
Multivariable model 2	1 (Reference)	0.79 (0.53 to 1.18)	0.73 (0.43 to 1.23)	0.318
Multivariable model 3	1 (Reference)	0.79 (0.53 to 1.18)	0.75 (0.44 to 1.27)	0.369

One serving of nuts equals 28 g. Cox regression models were used to assess the risk of cancer mortality by frequency of nut consumption. Multivariable model 1 was adjusted for age (years), sex, and intervention group. Model 2 was additionally adjusted for BMI (kg/m^2^), smoking status (never, former, current smoker), educational level (illiterate/primary education, secondary education, academic/graduate), leisure time physical activity (MET-min/day), history of diabetes (yes/no), history of hypercholesterolemia (yes/no), use of oral antidiabetic medication (yes/no), use of antihypertensive medication (yes/no), use of statins (yes/no), and total energy intake (kcal/day). Model 3 was also adjusted for dietary variables in quintiles (vegetables, fruits, red meat, eggs, and fish), alcohol intake (continuous, adding a quadratic term) and Mediterranean diet adherence (13-point score). All models were stratified by recruitment centre. Extremes of total energy intake were excluded.

**Figure 1:**
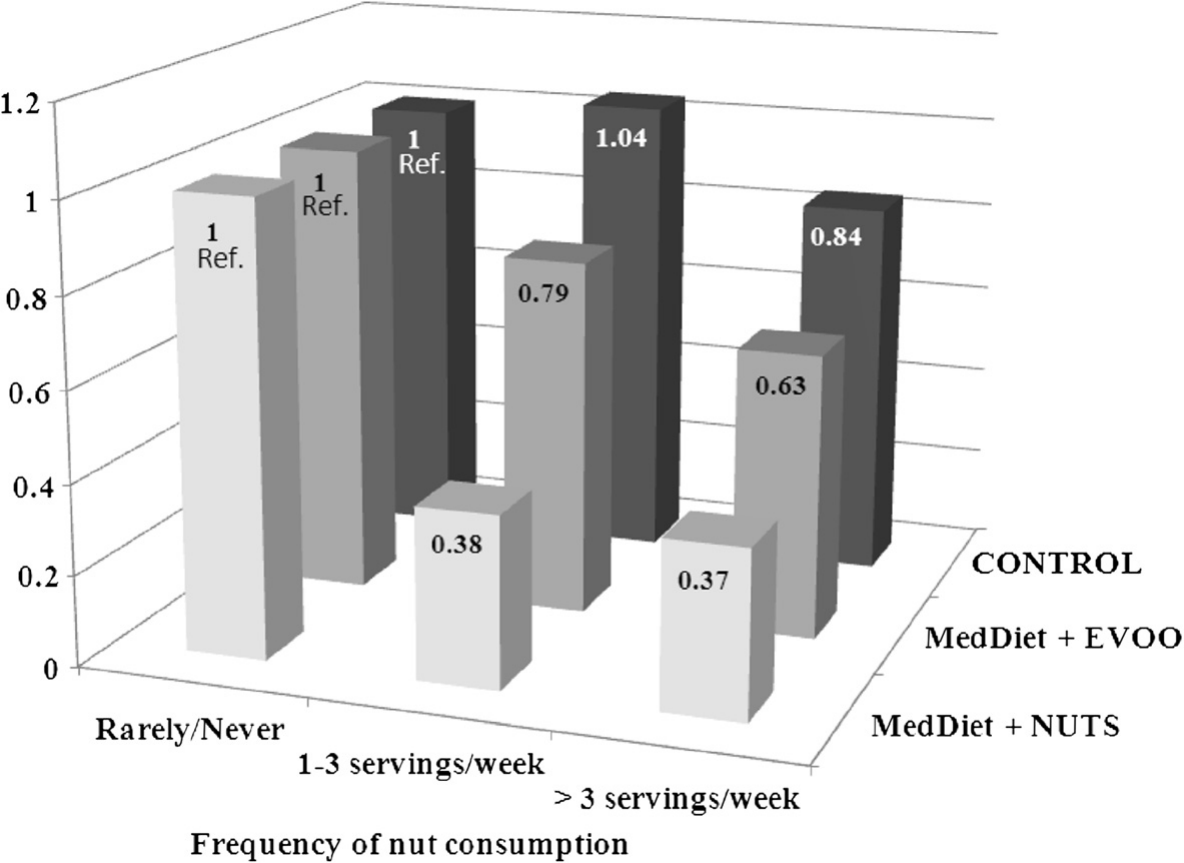
**Adjusted hazard ratios of total mortality by frequency of nut consumption and intervention group.** The Cox regression models were adjusted for age in years, sex, BMI in kg/m^2^, smoking status (never, former, current smoker), educational level (illiterate/primary education, secondary education, academic/graduate), leisure time physical activity in MET-min/day, history of diabetes (yes/no), history of hypercholesterolemia (yes/no), use of oral antidiabetic medication (yes/no), use of antihypertensive medication (yes/no), use of statins (yes/no), total energy intake (kcal/d), dietary variables in quintiles (vegetables, fruits, red meat, eggs and fish), alcohol intake (continuous, adding a quadratic term), and Mediterranean diet adherence (13-point score). The model was stratified by recruitment centre. Extremes of total energy intake were excluded. Values for the two upper categories of nut consumption are 0.38 (95% CI: 0.23 to 0.63) and 0.37 (95% CI: 0.22 to 0.66) in the Mediterranean diet supplemented with nuts (MedDiet + NUTS) group; 0.79 (95% CI: 0.50 to 1.24) and 0.63 (95% CI: 0.36 to 1.1) in the Mediterranean diet supplemented with extra-virgin olive oil (MedDiet + EVOO) group; and 1.04 (95% CI: 0.64 to 1.69) and 0.84 (95% CI: 0.48 to 1.44) in the low-fat control diet group. P for the interaction between baseline nut consumption and intervention group= 0.019. P for trend: MedDiet + NUTS, p=0.01; MedDiet + EVOO, p=0.15; Control diet, p=0.42.

Figure 1 shows the multivariate adjusted HRs for total mortality by frequency of total nut consumption and intervention group. In the three arms of the trial, individuals who consumed nuts >3 servings per week tended to have a lower risk of mortality than those in the reference category. Subjects in the upper category of nut consumption at baseline allocated to the MedDiet with nuts intervention had a significant reduction in total mortality risk of 63% (95% CI −34% to −78%), while those allocated to the MedDiet with EVOO and the control diet had non-significant reductions of 34% (95% CI −64% to 10%) and 16% (95% CI −52% to 44%), respectively. The interaction between baseline total nut consumption and intervention group was significant, *P* = 0.019).

When we used generalized estimating equations to assess the association between yearly updated measurements of total nut consumption and all-cause mortality we also found a significant inverse association. The fully-adjusted relative risk (RR) was 0.68 (95% CI 0.50 to 0.93) with a significant linear trend test. When we repeated the analysis to evaluate the association between nut intake and cardiovascular mortality and cancer mortality the fully-adjusted relative risk (RR) were 0.76 (95% CI 0.42 to 1.36) and 0.63 (95% CI 0.39 to 1.03), respectively; however the linear trend tests were not significant (data not shown).

## Discussion

In this longitudinal cohort study of individuals at high cardiovascular risk with relatively high nut intake living in a Mediterranean country, the frequency of nut consumption was inversely related to total mortality after 4.8 years of follow-up. Compared to non-consumers, subjects who consumed >3 servings of nuts per week at baseline had a significant 39% lower risk of all-cause mortality. Relative risk reductions were similar for the upper baseline category of non-walnut nuts (34%) or walnut consumption (45%), and when we evaluated the repeated measurements of total nut consumption over time (32%).

Moreover, those in the upper category of baseline nut consumption had a 55% lower risk of cardiovascular mortality and 40% lower risk of cancer mortality compared to those who never consumed nuts. The study subjects participated in the PREDIMED study, a long-term, randomized nutrition intervention trial [14], and those consuming more nuts at baseline and allocated to intervention with a MedDiet supplemented with nuts showed a significantly reduced total mortality risk of 63%.

The inverse association between baseline nut consumption and total mortality was of borderline significance in participants in the upper category of baseline nut consumption in a MedDiet supplemented with EVOO group, while there was no significant association in those allocated to a low-fat control diet, who were advised to reduce intake of all fatty foods, including nuts. Thus, advice against eating nuts throughout the study might have counterbalanced the protective effect of a lifetime intake of these foods. We assume that the baseline assessment can be considered as a good correlate of lifetime habits in this population.

Our findings concur with those of epidemiological studies showing inverse associations between nut consumption and cardiovascular mortality. Four large prospective studies have reported consistent inverse associations between nut consumption and fatal CHD or sudden cardiac death [23]–[26]. In the Adventist Health Study, subjects who consumed nuts >5 times per week had a 48% reduced risk of fatal CHD [23]. The reduction in death from CHD among women who consumed nuts 2 to 4 times/week in the Iowa Women’s Health Study were 57% [24]. However, a later report from the same study with longer follow-up failed to confirm that nut consumption protected from CHD death [10]. In addition, the Nurses’ Health Study observed that women who consumed nuts ≥5 times/week had a 30% reduced risk of fatal CHD [25]. Finally, the Physicians’ Health Study reported a 47% lower risk of sudden cardiac death and 30% lower risk of total CHD death among men who consumed nuts twice a week or more [7].

In our study, a reduced risk of cancer mortality has been observed in individuals that frequently consumed total nuts and walnuts. Few epidemiologic studies have been conducted evaluating the association between nut consumption and cancer. An ecological study showed that prostate cancer mortality was inversely associated with nuts and oilseed consumption [27]. Moreover, findings from prospective studies suggest inverse associations between nut consumption and colorectal or endometrial cancer, especially in women [28]–[31]. Some studies showed inverse associations of nut intake and prostate cancer [32], however the relationship between nuts and cancer incidence and mortality is insufficient and further research is needed [33]. A possible explanation that may account for the inverse relationship between walnuts and cancer mortality but not with other nuts could be that walnuts were richer in free and total polyphenols than all the other nuts [34]. As walnuts are usually consumed raw, and roasting can cause a decline in the efficacy in the antioxidant capacity, it has been shown that raw walnuts, as consumed in the PREDIMED study, had the highest antioxidant efficacy among all the nuts [34]; this could play a beneficial role in the prevention of cancer.

The present results also support those of prior observational studies suggesting that nut consumption protects against mortality. In the Iowa Women’s Health Study, subjects consuming nuts ≥2 times/week had a significant 12% lower mortality risk than those who ate nuts less than once monthly after a 12-year follow-up [10]. A recent study from a large Dutch cohort followed for 10 years reported that men and women in the 75th percentile of nut intake had 8% and 5% lower risks of all-cause mortality, respectively, compared with subjects in the 25th percentile [11]. Additionally, data from the Nurses’ Health Study, where participants were followed-up for nearly 18 years, showed that consuming nuts ≥2 times/week was associated with a 14% reduced risk of all-cause mortality [12]. It is noteworthy that the protection against total mortality afforded by nut consumption in our study was ≥3 orders of magnitude higher than that observed in studies of non-Mediterranean populations. A likely reason is that PREDIMED participants had a rather high self-selected nut intake before entering the study. Thus, 32% of PREDIMED participants consumed nuts >3 times/week, compared with nearly 10% consuming nuts ≥2 times/week in both the Iowa Women’s Health Study [10] and the Nurses’ Health Study [12]. In the Dutch study, participants in the 75th percentile of nut consumption had rather low average daily intakes of 11.1 g for men and 6.2 g for women [11].

The healthy nutritional profile of nuts may account for the inverse association observed between nuts and mortality. Nuts are high in monounsaturated fatty acids, fiber, minerals, vitamins and many bioactive compounds; all these nutrients may partly explain the beneficial effects on health that nuts have been shown to exert [3,4]. The frequency of nut consumption has been inversely related to several chronic prevalent conditions, such as diabetes, hyperlipidemia, hypertension, obesity, metabolic syndrome, cancer, and CHD, among others [5,26]. These inverse associations can be influenced by various mechanisms: nuts improve the blood lipid profile [6] and appear to decrease insulin resistance [8], and there is also evidence suggesting that they can modulate inflammation [35], oxidative stress [36], and endothelial function [37]. As a large body of evidence supports the beneficial effects of frequent nut consumption on many health outcomes, it is plausible that nuts protect as well against all-cause mortality.

Our study has limitations. First, given its observational nature, it is not possible to firmly conclude that the inverse relationship between nut consumption and total mortality reflects cause and effect. Second, even though data were adjusted for all possible confounders, there is still the possibility of residual confounding. However, the enhanced protective effect against all-cause mortality observed in frequent nut consumers at baseline who continued eating nuts during follow-up because they were allocated to the nuts intervention arm supports a causal relationship between increasing dietary exposure to nuts and reduced mortality. However, as the study was conducted in an older Mediterranean population at high cardiovascular risk, the results cannot easily be extrapolated to the general population. Nevertheless, it is relevant to assess these associations in individuals at high cardiovascular risk because this population is the most frequently attended by primary care physicians and the segment of population that can obtain higher benefits with diet or lifestyle changes.

There are also strengths to our study, such as a large sample size, relatively long duration of follow-up, and objective and thorough ascertainment of mortality as outcome in this prospective observational assessment.

## Conclusions

In summary, this study provides further evidence of the inverse relationship between the frequency of nut consumption and the risk of mortality in a Mediterranean population at high cardiovascular risk with relatively high nut intake.

## Appendix: other PREDIMED Investigators

Hospital Clinic, Institut d’Investigacions Biomediques August Pi i Sunyer, Barcelona, Spain: M Serra-Mir, A Pérez-Heras, C Viñas, R Casas, LS Romero, M Cofán, C Valls-Pedret, A Sala-Vila and M Doménech.

University of Navarra, Primary Care Centres, Pamplona, Spain: E Toledo, A Sánchez-Tainta, I Zazpe, M Marques, E Goñi, B Sanjulian, A Marti, P Buil-Cosiales, M Serrano-Martinez, J Diez-Espino, A Garcia-Arellano and FJ Basterra-Gortari.

University Rovira i Virgili, Reus, Spain: R Gonzalez, C Molina, F Marquez, N Babio, P Martinez, N Ibarrola-Jurado, R Balanza, A Díaz-López, M Juanola-Falgarona, M Sorlí, J Garcia Roselló, F Martin, R Tort, A Isach, B Costa, JJ Cabré and J Fernandez-Ballart.

Institut de Recerca Hospital del Mar, Barcelona, Spain: MI Covas, H Schröder, S Tello, R de la Torre, MA Muñoz and J Vila.

University Hospital of Alava, Vitoria, Spain: I Salaverría, S Castro, E Sanz, F Ricarte and J Rekondo.

University of Málaga, Málaga, Spain: R Benítez-Pont, M Bianchi-Alba, J Fernández-Crehuet and E Gómez-Gracia.

Department of Family Medicine, Primary Care Division of Sevilla, Sevilla, Spain: FJ García, M Ortega-Calvo, P Román, JM Santos and Y Corchado.

University of Las Palmas de Gran Canaria, Las Palmas, Spain: J Álvarez-Pérez, E Díez-Benítez, I Bautista-Castaño and A Sánchez-Villegas.

University of Valencia, Department of Preventive Medicine, Spain: C Ortega-Azorin, EM Asensio-Márquez, P Guillem-Saiz, JI Gonzalez and O Portoles.

## Abbreviations

CHD: Coronary heart disease; CVD: Cardiovascular disease; EVOO: Extra-virgin olive oil; MedDiet: Mediterranean diet.

## Competing interests

JS-S has received grants from Nut and Dried Fruit Foundation and is a non-paid member of the Scientific Advisory Board of the International Nut and Dried Fruit Foundation. ER has received grants from the California Walnut Commission and is a non-paid member of its Scientific Advisory Committee. No potential conflicts of interest relevant to this article were reported for any of the other authors. None of the funding sources played a role in the design, collection, analysis, or interpretation of the data or in the decision to submit the manuscript for publication.

## Authors’ contributions

MAM-G, DC, ER, RE, MIC, FA, JW, JL, MAM, RML-R, LS-M, XP, and JS-S designed research; MG-F, MB, MAM-G, DC, ER, RE, MIC, FA, JW, JL, MAM, RML-R, LS-M, XP, JB, JS-S conducted research; M-GF, MAM-G, and JS-S analyzed data; MG-F and JS-S wrote the paper; MAM, DC, RE, FA, JW, JL, MAM-G, LS-M, XP, and JS-S were the coordinators of subject recruitment at the outpatient clinics and MG-F and JS-S had primary responsibility for final content. All authors revised the manuscript for important intellectual content, read and approved the final manuscript.

## Pre-publication history

The pre-publication history for this paper can be accessed here:

http://www.biomedcentral.com/1741-7015/11/164/prepub
